# Photosynthetic Behavior of Wheat in Reclaimed Fly Ash Amended Soil—Probed by MINI‐PAM (Photosynthetic Yield Analyzer)

**DOI:** 10.1002/pei3.70143

**Published:** 2026-06-25

**Authors:** Chandralekha Piparia, Anjali Verma, Santosh Kumar Prajapati

**Affiliations:** ^1^ Department of Botany School of Life Sciences, Guru Ghasidas Vishwavidyalaya Chhattisgarh India

**Keywords:** chlorophyll fluorescence, heavy metals, MINI‐PAM, photosynthetic yield, *Triticum aestivum*

## Abstract

Fly ash (FA) management is a global environmental concern. Although FA has been widely studied as a soil amendment to improve crop growth and productivity, its utilization remains limited due to the presence of toxic heavy metals (HMs). Reclamation of FA using tolerant plant species is a sustainable strategy to mitigate adverse environmental effects and improve its suitability as a soil mulcher. However, studies evaluating the use of reclaimed fly ash (RFA) as a soil amendment remain limited. In the present study, wheat was cultivated under six conditions: T1 (normal soil, NS; control), T2 (fresh fly ash, FFA), T3 (RFA), and mixtures of RFA and NS at ratios of 1:1 (T4), 1:2 (T5), and 1:3 (T6). Prior to sowing, 12 targeted HMs were quantified using inductively coupled plasma–mass spectrometry (ICP‐MS). To assess the photosynthetic behavior of wheat, chlorophyll fluorescence parameters, including Fv/fm (variable to maximum chlorophyll fluorescence), Y(II) (effective quantum yield of photosystem II), Y(NPQ) (quantum yield of regulated non‐photochemical energy dissipation), and Y(NO) (quantum yield of non‐regulated energy dissipation), were measured with a pulse‐amplitude‐modulated chlorophyll fluorometer (MINI‐PAM). Results showed that Fv/fm and Y(II) were highest in T1, followed by T6, whereas Y(NPQ) and Y(NO) were lowest in T6, followed by T1. Spearman's correlation analysis revealed that Cr (chromium), Mn (manganese), Ni (nickel), Cu (copper), and Zn (zinc) were positively correlated with Fv/fm and Y(II) (*p* < 0.01), while As (arsenic), Se (selenium), and Mo (molybdenum) showed significant negative correlations (*p* < 0.001); opposite trends were observed for Y(NPQ) and Y(NO). Although heavy metal concentrations remained below established critical thresholds, even sub‐threshold variations influenced physiological performance. Overall, these findings highlight the potential of reclaimed fly ash as a soil amendment for sustainable wheat cultivation.

## 1 Introduction

Wheat ( *Triticum aestivum* L.) is the second most valuable cereal crop grown worldwide. In 2024, approximately 799 million tonnes (Mt) of wheat were produced globally across 220.7 million hectares. It remains a crucial dietary staple in numerous regions, providing approximately 20% of the global population's daily protein intake. Its ability to grow across diverse soils and climatic conditions significantly contributes to its widespread consumption (Food and Agriculture Organization of the United Nations (FAO) 2024).

India and China continue to dominate both cultivated area and output for the 2023–2024 marketing year. China produced approximately 136.6 Mt. (18% of global production), followed by India at 113.3 Mt. (14.2%), with a projected record production of 117.5 Mt. in 2025 (United States Department of Agriculture, Foreign Agricultural Service 2023). The Global Hunger Index (2023) ranked India 111th out of 125 countries, indicating a “serious” level of hunger. Wheat production is highly vulnerable to climate change. Increases in temperature (approaching 42°C), altered precipitation patterns, and changes in solar radiation may affect kernel development, grain filling (Subedi and Khadka 2022), and flag leaf photosynthesis, a central process determining growth and productivity (Feng et al. 2014).

Fly ash (FA) is an industrial by‐product that poses a significant environmental threat. Disposal of FA in open fields, either dry or wet, is a major ecological concern, causing land degradation, increased surface temperature, groundwater contamination, air pollution, and damage to soil and crop health (Fulekar and Dave 1986; Jala and Goyal 2006; Pandey et al. 2010; Dwivedi and Jain 2014). Developing countries such as China and India are among the largest generators of FA, with relatively low utilization rates; consequently, substantial quantities are disposed of as waste, affecting extensive land areas (Das and Rout 2023). Increasing electricity demand in India further accelerates FA generation, making the proper management of FA an urgent priority (India Water Portal 2023).

In addition to toxic heavy metals (HMs), FA contains essential macro‐ and micronutrients required for plant growth. Several studies have demonstrated that FA, when applied at appropriate rates, can enhance photosynthetic productivity in various crops. For example, a 25% FA amendment increased biomass, photosynthetic rate, and stomatal conductance in black gram (Panda, Mandal, et al. 2019; Panda, Sethy, et al. 2019). Chickpea exhibited increased chlorophyll content and antioxidant enzyme activity at FA concentrations up to 20% (Haris et al. 2021). Rice seedlings showed increased CO_2_ assimilation and antioxidant activity under 25% FA treatment while maintaining photosystem II function (Panda, Mandal, et al. 2019, Panda, Sethy, et al. 2019). In *Brassica juncea* , total chlorophyll content was highest at 30% FA application, whereas reductions were observed at 50% FA. Lower FA application rates enhanced crop performance, whereas higher doses adversely affected growth and productivity (Paul et al. 2024).

In wheat, 30% FA improved germination, whereas higher concentrations significantly inhibited growth (Liao et al. 2018; Vashistha and Tejasvi 2024). Excessive application of FA can alter soil characteristics, increase pH, reduce nutrient availability, and adversely affect crop physiology and productivity (Osán et al. 1996; Meravi and Prajapati 2014; Panda and Biswal 2018; Ahmad et al. 2021; Vashistha and Tejasvi 2024; Paul et al. 2024).

FA may also influence metal dynamics; while it can reduce the bioavailability of certain metals, it may increase the mobility of others, such as cadmium (Cd), depending on soil type, raising food safety concerns (Singh et al. 2024; Kumar et al. 2019; Wang et al. 2020). Therefore, FA reclamation is a prerequisite for its safe agricultural application. To date, limited studies have investigated phytoremediator species such as *Calotropis procera* , *Ipomoea carnea* , *Ricinus communis* , *Festuca rubra* , *Cassia tora* , and *Ziziphus nummularia* for FA reclamation (Gajić et al. 2018; Meravi and Prajapati 2019). HM‐ameliorated FA (i.e., reclaimed FA, RFA), with improved physicochemical properties, may serve as a soil mulch suitable for diverse soil types and has the potential to enhance agricultural productivity.

Although controlled pot experiments provide valuable insights into physiological responses under defined conditions, extrapolating these findings to field‐scale applications should be approached with caution. Soil heterogeneity, climatic variability, irrigation patterns, and the long‐term accumulation of HMs may influence the behavior of RFA in natural agricultural systems. In particular, the potential for HM leaching into deeper soil layers and groundwater remains an important environmental consideration under field conditions (Han et al. 2025; Mohamed et al. 2025; Xu et al. 2024; Sharafi and Saheli 2025). Furthermore, metal mobility and bioavailability can change over time depending on soil pH, organic matter content, and microbial activity (Zeng et al. 2011; Kicińska et al. 2022; Xu et al. 2022; Sheeba et al. 2024).

The present study evaluates the effect of RFA as a soil amendment on key chlorophyll fluorescence parameters—Fv/Fm (ratio of variable to maximum chlorophyll fluorescence), Y(II) (effective quantum yield of photosystem II), Y(NPQ) (quantum yield of regulated non‐photochemical energy dissipation), and Y(NO) (quantum yield of non‐regulated energy dissipation) measured in the flag leaf of wheat ( *Triticum aestivum* L., cv. Sarbati‐366). While the findings provide evidence of the short‐term physiological responses and immediate agronomic potential of RFA under controlled conditions, comprehensive long‐term field trials across diverse soil types and agro‐climatic regions are essential to fully assess its ecological sustainability, metal stability, and practical feasibility for large‐scale agricultural application.

## 2 Materials and Methods

### 2.1 Experimental Design

A completely randomized pot experiment (30 × 20 cm) was conducted using *Triticum aestivum* L. (cv. Sarbati‐366) under the shaded conditions in the Botanical Garden of Guru Ghasidas Vishwavidyalaya, Bilaspur, Chhattisgarh, India (22.1279° N, 82.1388° E). During the experimental period, November to February (2023–2024), the average temperature was 21°C ± 3°C, and the mean rainfall was 13 ± 4 mm.

### 2.2 Soil and FA Collection

FFA (fresh FA), collected from sites without plantations, and RFA, collected from sites with plantations, were obtained from the FA dyke near the NTPC Sipat Thermal Power Plant in Bilaspur, Chhattisgarh, India (latitude 22.89067° and longitude 82.292485°). Normal soil (NS) was collected from the Botanical Garden by digging to a depth of 5–10 cm. All samples were air‐dried and homogenized before use.

### 2.3 Growing Conditions

Six growing conditions were prepared on a weight‐to‐weight (w/w) basis, each in triplicate (total 18 pots). The conditions were: T1: NS as control, T2: FFA, T3: RFA, T4: RFA + NS (1:1), T5: RFA + NS (1:2), T6: RFA + NS (1:3). The mixture was thoroughly homogenized before filling the pots to ensure uniform distribution.

### 2.4 Physicochemical Characterization and Nutrient Status of Growing Parameters

The six growing conditions (T1—T6) were characterized for their physicochemical properties and nutrient status prior to plant growth assessment. pH and electrical conductivity (EC) were determined in a 1:2.5 (w/v) medium‐to‐distilled water suspension using a digital pH and conductivity meter, following the standard procedures described by Jackson (1973) and Page et al. (1982). Water‐holding capacity (WHC) was measured gravimetrically, as outlined by Keen and Raczkowski (1921). Bulk density (BD) was determined by the core method, and particle density (PD) was measured using the pycnometer method, following Black (1965). Total porosity (%) was calculated from BD and PD using the formula:

$$\text{Porosity}\;\left(\%\right)=\left(1-\frac{\mathrm{BD}}{\mathrm{PD}}\right)\times 100$$

Organic carbon (OC) content was determined by the Walkley and Black wet oxidation method (Walkley and Black 1934). Available nitrogen (N) was estimated using the alkaline permanganate method described by Subbiah and Asija (1956). Available phosphorus (P) was determined by the Olsen extraction method (Olsen et al. 1954), and available potassium (K) was analyzed by flame photometry after neutral ammonium acetate extraction, as described by Jackson (1973). Total micronutrients Copper (Cu), Boron (B), and Zinc (Zn) were determined after acid digestion and quantified by Inductively Coupled Plasma Mass Spectrometry (ICPMS‐Agilent; 7900), following the procedures described by Lindsay and Norvell (1978).

### 2.5 Heavy Metal Analysis

The six prepared growing conditions (T1–T6) were analyzed for twelve targeted HMs, namely Cd, Cr (chromium), Mn (manganese), Ni (nickel), Cu (copper), Zn (zinc), As (arsenic), Se (selenium), Mo (molybdenum), Pb (lead), Fe (iron), and Co (cobalt). Samples were digested using concentrated HNO_3_ prior to quantification by Inductively Coupled Plasma Mass Spectrometry (ICP–MS; Agilent 7900).

### 2.6 Planting and Growth Conditions

Seeds ( *Triticum aestivum* L.) were soaked overnight prior to sowing to ensure uniform germination. 15 seeds were sown in each pot under the respective growing conditions. Two weeks after germination, seedlings were thinned to maintain uniform plant density and growth across conditions. Pots were watered daily as required to maintain adequate soil moisture throughout the experimental period.

### 2.7 Photosynthetic Yield Estimation

Photosynthetic parameters were measured with a pulse‐amplitude‐modulated chlorophyll fluorometer (MINI‐PAM; Heinz Walz GmbH, Germany). The parameters calculated were Fv/Fm = (Fm −Fo)/Fm, Y(II) = (Fm′−F′)/Fm′, Y(NPQ) = 1 − Y(II) − Y(NO), and Y(NO), following standard chlorophyll fluorescence protocols. The instrument comprises 51 single‐ and double‐key operations and can be operated manually or via the WinControl‐3 software designed for chlorophyll fluorometers.

Fully expanded flag leaves were selected and dark‐acclimated for 20–30 min using DLC‐8 magnetic leaf clips before measurement. After dark acclimation, the fiber‐optic probe was placed in the leaf clip holder and connected to the main control unit and the RS‐232 PC interface for data acquisition. Minimum fluorescence (Fo) was recorded under weak modulated measuring light, and maximum fluorescence (Fm) was then determined with a saturating light pulse. Actinic light was applied to obtain steady‐state fluorescence (F′) and maximum fluorescence in the light‐adapted state (Fm′). All measurements were made with the internal red LED light source (650 nm). Fluorescence data were saved and exported to an Excel spreadsheet for subsequent analysis.

### 2.8 Statistical Analysis

All HM data were reported as the mean ± standard deviation (SD) from three replicates. Statistical analyses were performed using SPSS version 20.0 (IBM Corp., Armonk, NY, USA). Normality was assessed with the Shapiro–Wilk test. Differences in HM content among treatments were analyzed by one‐way analysis of variance (ANOVA), and the relationship between HM concentrations and photosynthetic parameters was examined using Spearman's correlation test.

## 3 Results

The physicochemical characteristics and nutrient status of the six growth conditions (T1–T6) are presented in Table 1. Across all treatments, soil pH remained slightly alkaline (7.2–7.8), and EC values were consistently low (0.54–0.62 dS m^−1^), indicating non‐saline conditions suitable for plant growth. WHC decreased substantially from T1 to T2 (46%) but increased progressively from T4 to T6, reaching the highest value at T6 (69%). BD decreased from 1.25 g cm^−3^ in T2 to 0.76 g cm^−3^ in T4, while PD remained relatively stable (2.2–2.9 g cm^−3^) across treatments, resulting in corresponding changes in porosity.

**TABLE 1:** Physicochemical properties and nutrient status of all the growing conditions (T1‐T6).

Physico‐chemical parameters	Growing conditions					
	T1 (NS)	T2 (FFA)	T3 (RFA)	T4 (1:1)	T5 (1:2)	T6 (1:3)
Texture	Sandy loam	Silty	Silty loam	Loam	Sandy loam	Sandy loam
pH	7.2 ± 2	7.8 ± 2	7.3 ± 2	7.5 ± 2	7.4 ± 2	7.3 ± 2
EC (dS m^−1^)	0.541	0.62	0.581	0.561	0.553	0.547
WHC (%)	65	53.04	62	63	66	69
BD (g/cm^3^)	1.12	1.25	0.92	0.76	0.85	0.96
PD (g/cm^3^)	2.27	2.93	2.24	2.24	2.21	2.25
Porosity (%)	50.7	57.3	59.9	54.2	53.9	53.3
OC (%)	0.65	0.03	0.07	0.29	0.42	0.49
Available P (mg kg^−1^)	10.25	4.8	2.4	4.2	5.71	4.12
Available N (mg kg^−1^)	100.2	ND	ND	47.6	52.9	55.3
Available K (mg kg^−1^)	187.5	154.12	136.14	157.5	162.75	175.91
Total Cu (mg kg^−1^)	39.92	16.43	15.19	27.56	33.72	34.16
Total Zn (mg kg^−1^)	41.12	110	87	62.37	58.15	55.67
Total B (mg kg^−1^)	8.6	30	20.01	13.98	13.62	11.65

*Note:* T1 (NS—normal soil), T2 (FFA—fresh fly ash), T3 (RFA—reclaimed fly ash), and mixtures of RFA and NS at ratios of 1:1 (T4), 1:2 (T5), and 1:3 (T6). All nutrient concentrations are expressed in mg kg^−1^ (dry weight basis).

Abbreviations: BD, bulk density; EC, electrical conductivity; ND, not detected; OC, organic carbon; PD, particle density; WHC, water holding capacity.

Nutrient status varied considerably among treatments. OC was considerably lower in T2 and T3 compared to T1 (0.65%); similarly, available P, N, and K declined sharply in T2 relative to T1, with nitrogen not detected (ND) in T2 and T3. In contrast, micronutrients such as total Zn and B were higher in T2 compared to T1. In conditions T4–T6, physicochemical properties improved progressively. OC and N partially recovered, with T6 showing the greatest improvement. In contrast, T2 and T3 exhibited lower fertility and poorer physical structure, whereas T5 and T6 provided more balanced and favorable conditions.

Concentrations of HMs under all six growing conditions (T1–T6) are listed in Table 2. Concentrations of Cr, Mn, Ni, Cu, and Zn were significantly lower in T2 and T3 than in T1, with intermediate values in the other three conditions (T4, T5, T6). This suggests reduced availability of these HMs in FA‐amended soils. Co and Pb were lower in T3 than in T2 and in the other amendments. Likewise, the other HMs, As, Se, and Mo, showed maximum mean concentrations in T2. Fe remained relatively stable across all five growing conditions (T2–T6) compared to T1. Cd did not change significantly across treatments. One‐way ANOVA confirmed that concentrations of all HMs differed significantly among treatments (*p* < 0.05), whereas Cd did not differ significantly (*p* > 0.05).

**TABLE 2:** Concentration (mg kg^−1^) of targeted metals in six growing conditions.

Growing amendments	Cd	Cr	Mn	Co	Ni	Cu	Zn	As	Se	Mo	Pb	Fe
T1 (NS)	0.33 ± 0.10 ns	64.62 ± 0.58*	767.74 ± 9.41*	7.41 ± 0.46*	33.63 ± 2.03*	45.44 ± 0.48*	78.64 ± 1.08*	5.69 ± 0.06*	0.60 ± 0.06*	2.37 ± 0.17*	12.34 ± 1.11*	544.67 ± 8.96*
T2 (FFA)	0.37 ± 0.06 ns	44.82 ± 0.55*	199.12 ± 0.61*	7.17 ± 1.00 *	21.36 ± 0.63*	16.29 ± 0.26*	42.92 ± 1.63*	13.80 ± 0.69 *	6.13 ± 0.19*	4.34 ± 0.12*	15.66 ± 2.93*	850 ± 64.86*
T3 (RFA)	0.29 ± 0.06 ns	40.53 ± 0.57*	132.81 ± 1.02*	4.10 ± 0.11*	13.89 ± 0.56*	15.17 ± 0.02*	24.57 ± 0.15*	6.99 ± 0.70*	2.35 ± 0.03*	4.00 ± 0.69*	8.87 ± 1.08*	687.33 ± 10.50*
T4 (1:1)	0.42 ± 0.04 ns	54.04 ± 1.26*	441.22 ± 5.76*	5.84 ± 0.13*	24.05 ± 1.01*	32.03 ± 2.12*	51.64 ± 0.22*	6.12 ± 0.02*	1.72 ± 0.34*	3.78 ± 0.67*	11.33 ± 0.91*	678.13 ± 8.23*
T5 (1:2)	0.37 ± 0.05 ns	58.25 ± 0.55*	550.38 ± 0.97*	6.39 ± 0.96*	28.33 ± 0.63*	35.24 ± 0.51*	64.44 ± 1.10*	5.51 ± 0.49*	1.40 ± 0.18*	2.56 ± 0.23*	11.66 ± 0.87*	724.67 ± 18.18*
T6 (1:3)	0.42 ± 0.02 ns	61.59 ± 0.69*	605.44 ± 6.81*	6.51 ± 0.55*	31.01 ± 0.10*	41.98 ± 2.40*	66.40 ± 0.76*	5.72 ± 0.19*	1.22 ± 0.25*	2.35 ± 0.36*	14.23 ± 1.55*	788.33 ± 73.82*

*Note:* All the data are represented as mean ± standard deviation (SD), *n* = 18. Level of significance tested using one way ANOVA. ns = *p* > 0.05. T1 (NS—normal soil), T2 (FFA—fresh fly ash), T3 (RFA—reclaimed fly ash), and mixtures of RFA and NS at ratios of 1:1 (T4), 1:2 (T5), and 1:3 (T6).

* *p* < 0.05.

**FIGURE 1:**
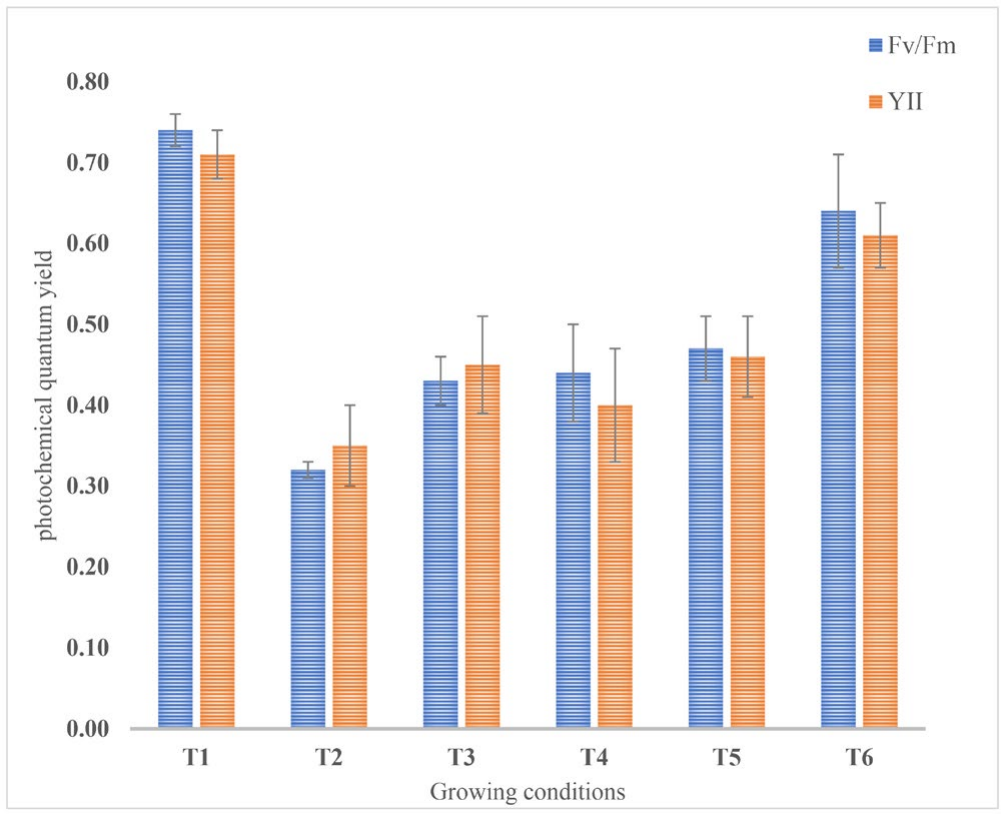
PSII efficiency (Fv/Fm) and effective quantum yield Y(II) of wheat grown under six growing conditions. T1 (normal soil, NS; control), T2 (fresh fly ash, FFA), T3 (reclaimed fly ash, RFA), and mixtures of RFA and NS at ratios of 1:1 (T4), 1:2 (T5), and 1:3 (T6). Data represent mean ± standard error (SE) of three biological replicates (*n* = 3). Error bars indicate the standard error of the mean.

**FIGURE 2:**
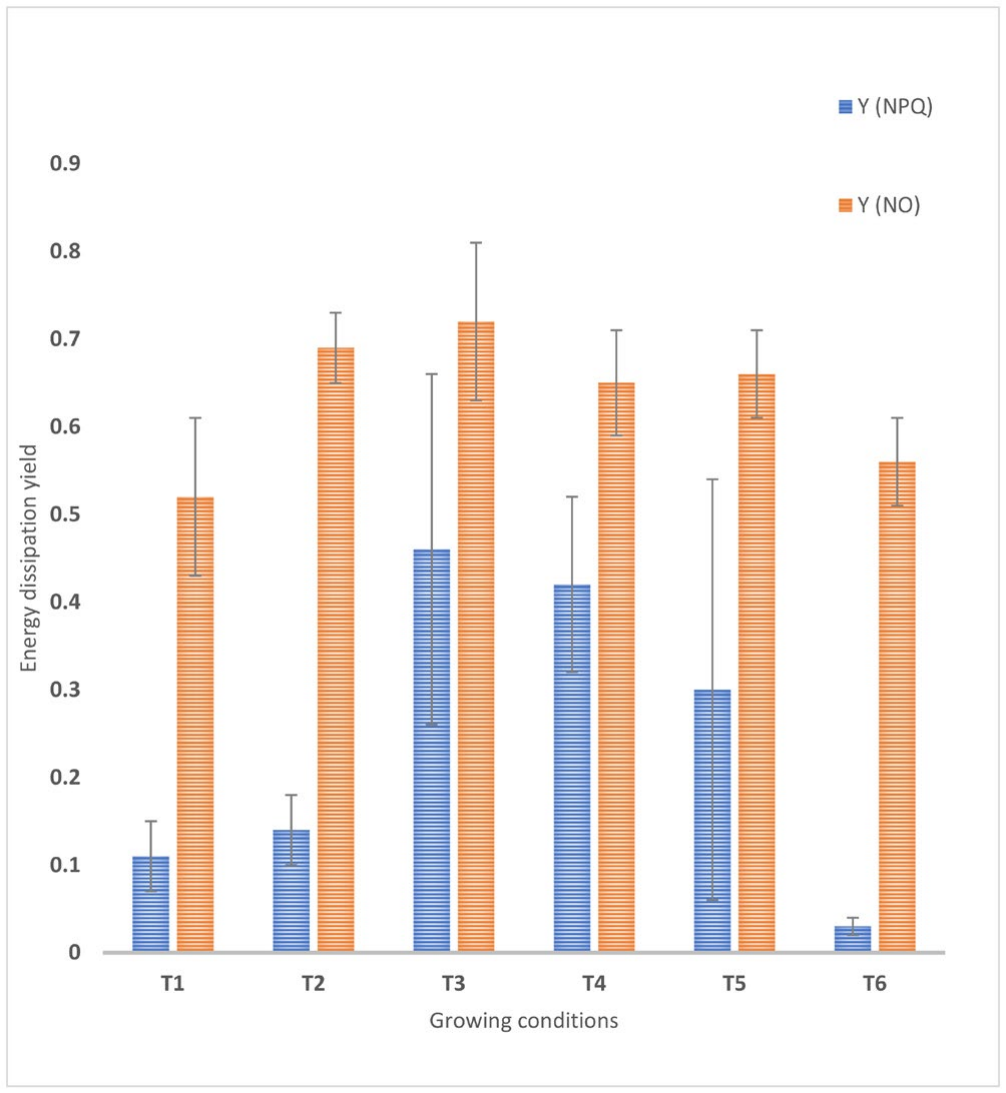
Non‐photochemical Y(NPQ) and non‐regulated Y(NO) energy dissipation of wheat grown under six growing conditions. T1 (normal soil, NS; control), T2 (fresh fly ash, FFA), T3 (reclaimed fly ash, RFA), and mixtures of RFA and NS at ratios of 1:1 (T4), 1:2 (T5), and 1:3 (T6). Data represent mean ± standard error (SE) of three biological replicates (*n* = 3). Error bars indicate standard error of the mean.

Photosynthetic parameters, including Fv/Fm, Y(II), Y(NPQ), and Y(NO), were measured in wheat grown under six conditions (T1–T6). T1 showed the highest chlorophyll fluorescence (Fv/Fm—0.74 and Y(II)—0.71), followed by T6 (0.64 and 0.61), while T2 showed the lowest values (Figure 1). Likewise, Y(NPQ) and Y(NO) were measured on the same day (Figure 2). The minimum Y(NPQ)—0.03 was recorded under T6, with a moderate decline in Y(NO)—0.56. In contrast, T3 showed the highest values under both conditions (0.46 and 0.72). T4 (0.42) and T5 (0.30) showed moderate Y(NPQ) activity, with relatively high Y(NO) values of 0.65 and 0.66, suggesting stress tolerance. Surprisingly, T1 (0.11) and T2 (0.14) had nearly equal Y(NPQ) values, though Y(NO) was higher in T2 (0.69) than in T1 (0.52). Variation in Y(NPQ) was relatively high among the parameters studied. Results showed that Y(NO) remained consistently higher than Y(NPQ) across all treatments.

To assess how HMs in the growth parameters affected the photosynthetic yield of wheat, Spearman's correlation test was performed (Table 3). Strong positive correlations were observed between the concentrations of Cr (*r* = 0.775 and 0.668), Mn (*r* = 0.757 and 0.699), Ni (*r* = 0.728 and 0.650), Cu (*r* = 0.804 and 0.678), and Zn (*r* = 0.749 and 0.625) and Fv/fm and YII, respectively (*p* < 0.01). Se (*r* = −0.782 and −0.687), Mo (*r* = −0.790 and −0.711), and R (*r* = −0.730 and −0.625) were negatively correlated (*p* < 0.001), indicating that treatment T2 strongly affected the photosynthetic mechanism. However, no significant relationships were observed between Cd, Pb, and Fe concentrations and the photosynthetic parameters (*p* > 0.05). On the other hand, Se and Mo were positively correlated with Y(NPQ) (*r* = 0.496 and 0.515, *p* < 0.05), and they elicit active dissipation of light energy to cope with stress. Se was significantly positively correlated with Y(NO) (*r* = 0.584, *p* < 0.05), whereas Mo showed a weak, non‐significant correlation (*r* = 0.220, *p* > 0.05). However, both Y(NPQ) and Y(NO) were negatively correlated with metals Cr, Cu, Zn, and Ni (*r* = −0.49 to −0.54, *p* < 0.05).

**TABLE 3:** Spearman's correlation matrix of analyzed heavy metals and chlorophyll fluorescence parameters.

Variable	Cd	Cr	Mn	Co	Ni	Cu	Zn	As	Se	Mo	Pb	Fe	Fv/Fm	YII	YNPQ	YNO
Cd	1.00															
Cr	0.20	1.00														
Mn	0.21	0.973**	1.00													
Co	0.16	0.538*	0.554*	1.00												
Ni	0.21	0.975**	0.986**	0.569*	1.00											
Cu	0.21	0.983**	0.963**	0.498*	0.967**	1.00										
Zn	0.23	0.959**	0.965**	0.511*	0.975**	0.975**	1.00									
As	−0.23	−0.767**	−0.802**	−0.19	−0.801**	−0.785**	−0.806**	1.00								
Se	−0.10	−0.902**	−0.903**	−0.26	−0.893**	−0.882**	−0.890**	0.776**	1.00							
Mo	−0.03	−0.813**	−0.822**	−0.28	−0.833**	−0.822**	−0.799**	0.875**	0.756**	1.00						
Pb	0.38	0.32	0.33	0.601**	0.32	0.35	0.33	−0.09	0.05	−0.25	1.00					
Fe	0.21	−0.27	−0.27	0.12	−0.27	−0.28	−0.31	0.23	0.510*	0.16	0.603**	1.00				
Fv/Fm	0.08	0.775**	0.757**	0.13	0.728**	0.804**	0.749**	−0.730**	−0.782**	−0.790**	0.13	−0.35	1.00			
YII	0.07	0.668**	0.699**	0.25	0.650**	0.678**	0.625**	−0.625**	−0.687**	−0.711**	0.14	−0.28	0.908**	1.00		
YNPQ	−0.10	−0.493*	−0.45	−0.10	−0.508*	−0.480*	−0.508*	0.38	0.496*	0.515*	−0.20	−0.09	−0.23	−0.07	1.00	
YNO	−0.14	−0.540*	−0.46	−0.14	−0.510*	−0.498*	−0.497*	0.30	0.584*	0.22	0.09	0.20	−0.30	−0.18	0.547*	**1.00**

*Note:* Values represent Spearman's correlation coefficients (*n* = 18).

* *p* ≤ 0.05.

** *p* ≤ 0.01 (two‐tailed test).

## 4 Discussion

FA amendment significantly influenced the physicochemical and nutrient characteristics of the growing conditions. Although pH remained slightly alkaline and EC values indicated non‐saline conditions suitable for plant growth, T2 exhibited reduced WHC, higher BD, and markedly lower OC and available macronutrients compared to T1. Such reductions in fertility parameters are consistent with previous reports describing the low organic matter and nitrogen status of FA‐dominant substrates (Jala and Goyal 2006; Dwivedi and Jain 2014). In contrast, micronutrients such as Zn and B were comparatively higher in T2 followed by T3, reflecting their mineral composition. Importantly, progressive amendment of RFA with soil (T4–T6) improved physical structure and partially restored OC and available N, with T6 exhibiting the most balanced conditions. These findings support earlier studies indicating that appropriate soil–FA ratios can mitigate structural and nutritional limitations and enhance their suitability for crop growth (Pandey et al. 2010; Shakeel et al. 2022).

FFA exhibited higher HM concentrations than RFA, indicating that phytoremediation substantially reduces metal load and improves suitability for agricultural use (Meravi and Prajapati 2019). Correspondingly, in wheat, the photosynthetic parameters Fv/Fm and Y(II) were highest in T1, followed by T6, whereas the lowest values were observed in T2. This pattern suggests that elevated HM concentrations in T2 interfered with photosynthetic electron transport and reduced PSII efficiency. The optimal Fv/Fm range for healthy plants is generally 0.75–0.85 (Bjorkman and Demmig 1987; Maxwell and Johnson 2000). While T1 remained within this optimal range, T6 recorded a value of 0.64, indicating moderate stress but comparatively better performance than the other FA‐amended treatments. In contrast, Y(NPQ) was highest at T3, followed by T4 and T5, reflecting activation of regulated non‐photochemical quenching as a protective mechanism for dissipating excess excitation energy as heat. Likewise, Y(NO) increased in the order T3 > T2 > T5 > T4 > T6 > T1, suggesting that plants exposed to higher HM levels relied more heavily on non‐regulated energy dissipation pathways, which are often associated with photo‐oxidative damage (Meravi and Prajapati 2020; Meravi et al. 2021).

Previous studies in other crops have reported that FA, when applied at lower rates, can enhance nutrient availability as a soil amendment by providing essential macro‐ and micronutrients like P, K, Ca, Mg and B; however, large‐scale application is often constrained by HM content (Pandey et al. 2010; Dwivedi and Jain 2014; Shakeel et al. 2022). The sensitivity to these HMs varies significantly across species. For instance, while rice ( *Oryza sativa* L.) exhibits significant phytotoxicity and 14–15 fold increases in metal accumulation in roots when grown in 50% FA (Singh et al. 2016), mustard ( *Brassica juncea* ) has shown the ability to accumulate toxic metals within WHO permissible limits even under higher amendment rates, suggesting a higher tolerance threshold for oilseed crops (Vashistha and Tejasvi 2024). The presence of HMs in the growing medium can indirectly influence nutrient dynamics and physiological processes, ultimately affecting photosynthetic efficiency in crops (Meravi and Prajapati 2014; Meravi et al. 2023; Vasilachi et al. 2023). Toxic metals such as arsenate [As(V)] disrupt phosphate metabolism, while Se, Ni, Cr, and Pb induce oxidative stress and damage thylakoid membranes, reducing photochemical quantum efficiency (Shri et al. 2019; Bashir et al. 2016). In legumes like mung bean ( *Vigna radiata* ) and urad bean ( *Vigna mungo* ), growth is maximized at relatively low FA concentrations (10%–20%), beyond which significant yield decline occurs (Dhadse 2024).

Comparatively, wheat in the present study exhibited moderate sensitivity. Unlike the rapid decline seen in legumes, wheat has been shown to tolerate FA as soil amendment up to 40%–60% before experiencing a sharp drop in germination and biomass, with some varieties even showing enhanced photosynthetic pigments at 40% application (Vashistha and Tejasvi 2024). In this study, although FFA induced stress, the use of reclaimed and their amendment with soil successfully mitigated the extent of this damage, consistent with recent findings that FA‐based soil conditioners can increase wheat yields by up to 15% in specific soil types (Ou et al. 2020).

To further evaluate the environmental safety of RFA application, HM concentrations across treatments were compared with internationally cited permissible limits for agricultural soils and Indian reference standards (Table S1). The maximum concentrations of Cd, Cr, Ni, Cu, Zn, As, Mo, and Pb remained below the commonly referenced threshold values reported by World Health Organization (WHO) and Food and Agriculture Organization (FAO) (1984) and Awasthi (2000). Although Se concentrations were highest in T2, reclamation and soil mixing substantially reduced Se levels in amended treatments, indicating stabilization and dilution. Although HM concentrations varied significantly across treatments, all values remained within their respective reference thresholds, indicating that wheat plants were not exposed to acute regulatory exceedance. However, the observed correlations between specific HM and chlorophyll fluorescence parameters demonstrate that even subcritical variations in HM concentrations can influence physiological performance. These findings highlight the sensitivity of chlorophyll fluorescence as an early indicator of metal‐induced stress and underscore the importance of integrating physiological assessment with regulatory benchmarking when evaluating RFA for sustainable agricultural applications. However, these findings are derived from pot‐scale experiments; research including plant tissue metal analysis and field evaluation is necessary to assess the long‐term agricultural effectiveness and environmental safety of RFA‐based amendments.

## 5 Conclusion

The present study shows that wheat grown in FA‐amended soils exhibits distinct physiological responses that depend on the FA proportion. FFA exhibited higher HM concentrations than RFA, underscoring the effectiveness of phytoremediation in reducing metal load. Although all targeted metal concentrations remained below their critical limits, variation among them significantly affected photosynthetic behavior. Treatments with higher metal content, particularly T2 and T3, showed reduced photochemical efficiency and increased non‐regulated energy dissipation, indicative of stress‐induced impairment. In contrast, T6 performed best among all treatments, with improved Fv/Fm and Y(II), suggesting that optimized RFA proportions can support higher plant productivity while minimizing metal‐induced stress. Overall, the findings confirm that RFA‐based soil amendments can be safely used for wheat cultivation without exceeding HM toxicity thresholds; however, subcritical metal levels still modulate photosynthetic efficiency. This underscores the need to optimize RFA‐soil ratios for sustainable crop production. These findings show that even when metal levels meet regulatory limits, plant physiology may still be affected, highlighting the value of chlorophyll fluorescence as an early indicator of stress. Nevertheless, field trials and tissue metal assessments are required to evaluate long‐term agronomic and environmental sustainability.

## Funding

The authors have nothing to report.

## Ethics Statement

The authors have nothing to report.

## Consent

The authors have nothing to report.

## Conflicts of Interest

The authors declare no conflicts of interest.

## Supporting information


**Table S1:** Critical threshold levels of selected heavy metals in agricultural soil.

## Data Availability

The data that support the findings of this study are included in the manuscript.
