# Supplementary material for: Photosynthetic Behavior of Wheat in Reclaimed Fly Ash Amended Soil—Probed by MINI‐PAM (Photosynthetic Yield Analyzer)
Source: Plant Environ Interact. 2026 Jun 25;7(3):e70143. doi: 10.1002/pei3.70143 (PMC13296575; doi:10.1002/pei3.70143)
Supplement: Supplementary file 1 — Table S1: Critical threshold levels of selected heavy metals in agricultural soil. [file PEI3-7-e70143-s001.docx]

| S. No. | Heavy metals  (mg kg^-1^) | WHO/FAO guideline for agricultural soil^a^ | USEPA^b^ | Indian standard for agricultural soil^c^ |
| --- | --- | --- | --- | --- |
| 1 | Cr | 100 | 11 | 100-120 |
| 2 | Mn | 2000 | 500 | - |
| 3 | Fe | - | - | 75-150 |
| 4 | Co | 50 | 3-200 | 60-110 |
| 5 | Ni | 50 | 72 | 75-150 |
| 6 | Cu | 100 | 4300 | 100-120 |
| 7 | Zn | 300 | 1100 | 300-600 |
| 8 | As | 20 | 75 | - |
| 9 | Mo | - | - | - |
| 10 | Cd | 3 | 0.48 | 3-6 |
| 11 | Pb | 100 | 200 | - |
| 12 | Se | - | - | - |

^a^ WHO/FAO guideline for agricultural soils (WHO/FAO 1984)

^b^ US Environmental Protection Agency (USEPA 2002)

^c^ Indian standards for agricultural soil (Awasthi 2000)

SUPPLEMENTARY TABLE

TABLE S1. Critical threshold levels of selected heavy metals in agricultural soil
